# Mid-term results of mitral valve repair using flexible bands versus complete rings in patients with degenerative mitral valve disease: a prospective, randomized study

**DOI:** 10.1186/s13019-017-0679-0

**Published:** 2017-12-13

**Authors:** Alexandr V. Bogachev-Prokophiev, Alexandr V. Afanasyev, Sergei I. Zheleznev, Vladimir M. Nazarov, Ravil M. Sharifulin, Alexandr M. Karaskov

**Affiliations:** Heart Valves Surgery Department, Meshalkin National Medical Research Center Ministry of Health Russian Federation, 15 Rechkunovskaya street, Novosibirsk, Russian Federation 630055

**Keywords:** Degenerative mitral valve disease, Mitral regurgitation, Mitral valve repair

## Abstract

**Background:**

We aimed to compare the outcomes of mitral valve repair with flexible band (FB) versus complete semirigid ring (SR) in degenerative mitral valve disease patients.

**Methods:**

From September 2011 to 2014, 171 patients were randomized and underwent successful mitral valve repair using a SR (*n* = 85) or FB (*n* = 86). There were no significant between-group differences at baseline.

**Results:**

There were no early mortalities. The mean follow up was 24.7 months. The 2-year survival was 96.0 ± 2.3% (95% confidence interval [CI], 88.6–98.7%) and 94.3 ± 2.8% (95% CI, 85.5–97.9%) in the SR and FB groups, respectively (*p* = 0.899). The left ventricle remodeling was similar between the groups. Higher transmitral peak (8.5 [3.9–17] vs. 6 [2.1–18] mmHg, *p* < 0.001), mean pressure gradients (3.7 [1.3–8] vs. 2.8 [0.6–6.8] mmHg, *p* = 0.001), and systolic pulmonary artery pressure (34.5 [20–68] vs. 29.5 [8–48] mmHg, p < 0.001) was observed in the SR group. The 2-year freedom from recurrence of significant mitral regurgitation was significantly higher in the FB group than the SR group (*p* = 0.002). Residual mitral regurgitation was an independent prognostic factor of recurrence of mitral regurgitation. The 3-year freedom from reoperation was significantly higher in the FB group than the SR group (*p* = 0.044).

**Conclusion:**

Patients with degenerative mitral valve disease may benefit from valve repair with FBs. Residual mitral regurgitation before discharge is an independent risk factor of late insufficiency recurrence.

**Trial registration:**

ClinicalTrials.gov NCT03278574, retrospectively registered on 06.09.2017.

**Electronic supplementary material:**

The online version of this article (10.1186/s13019-017-0679-0) contains supplementary material, which is available to authorized users.

## Background

In 1957, Lillehei et al. [1] proposed the mitral annuloplasty technique, a new concept in valve surgery. Remodeling annuloplasty, developed by Carpentier in 1983, increases leaflet coaptation, prevents future annular dilatation, and preserves leaflet mobility in patients with degenerative mitral valve (MV) disease [2, 3]. Currently, there are many commercially available mitral annuloplasty devices in the market, including complete or partial, rigid or flexible, and flat or saddle-shaped rings. However, no single annuloplasty device has been proven to have a clinical benefit above the others [4]. Theoretically, the flexible band does remodel mitral annulus providing reduction annuloplasty only. However, the rigid ring does not have enough flexibility for physiological annular motion during the cardiac cycle. A systematic review of clinical trials [5] showed comparable clinical outcomes between rigid and flexible rings. Currently, mitral ring selection is based on a surgeon’s preference rather than evidence [6]. Semirigid rings combine flexibility and stability; however, their clinical benefit has not been completely clarified. The present study aimed to compare the outcomes of MV repair with a flexible posterior annuloplasty band versus complete semirigid ring in patients with degenerative MV disease.

## Methods

### Study design

**Fig. 1:**
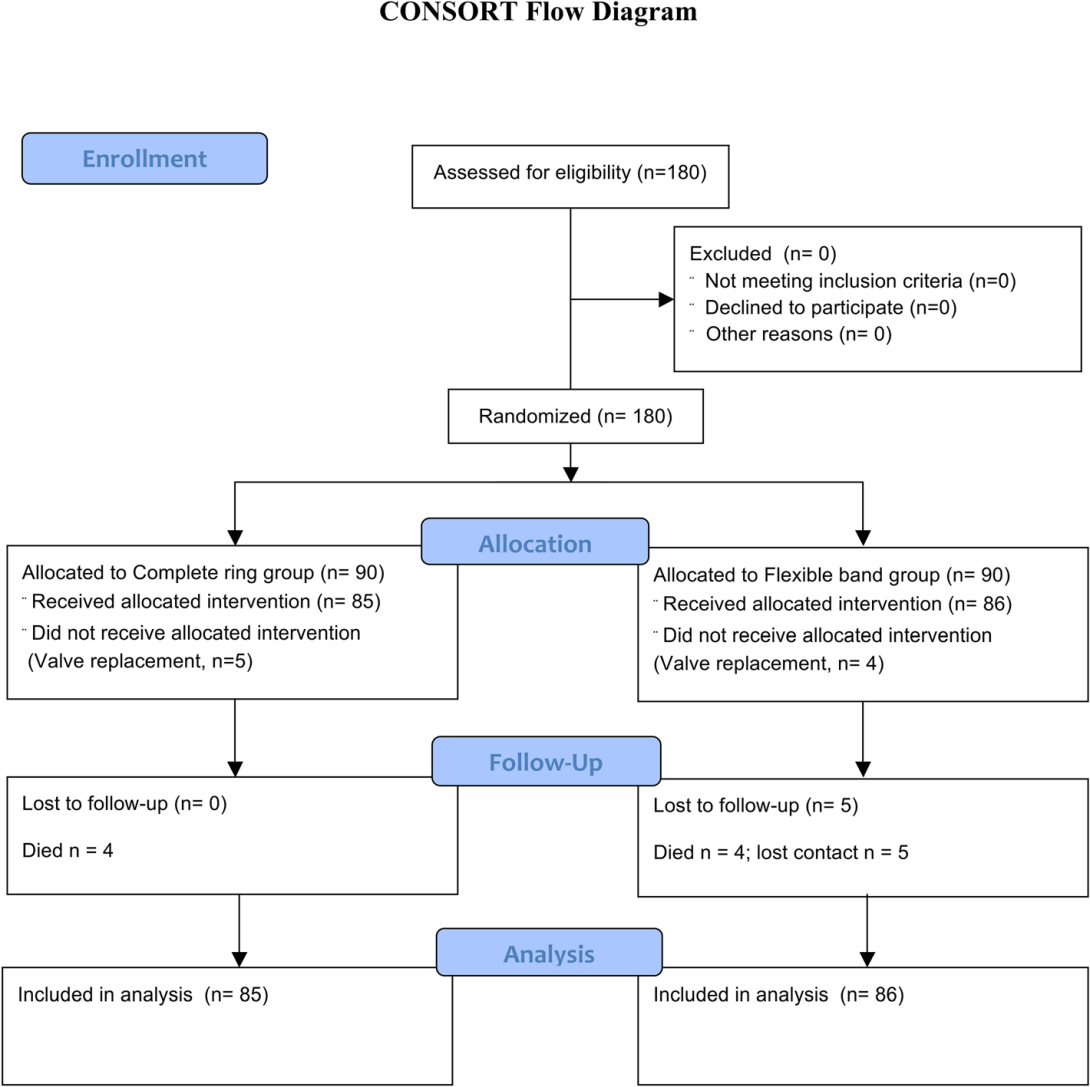
Consort flow diagram

In this prospective, randomized study, 171 patients with degenerative MV disease who were scheduled for isolated MV repair in our Institute from September 2011 through September 2014 were enrolled (CONSORT flow diagram, Figure 1). Participants were randomly assigned following a simple randomization procedure to complete semirigid ring (SR group) or flexible posterior annuloplasty band (FB group), according to computerized random numbers on the day before surgery. Eligible participants were adults aged 18 years or more with degenerative MV disease [7] who met the indications for MV operation according to the American College of Cardiology/American Heart Association guidelines [8]. Exclusion criteria were previous open cardiac surgery, indication for concomitant aortic valve replacement, or left ventricle (LV) impairment (ejection fraction <40%). The Local Ethics Committee approved the study design, and all the patients provided informed consent. The present study was conducted in compliance with the Declaration of Helsinki.

### Patients

Mean age of the participants in each group was 57 (23–75) and 54 (19–74) years, respectively. There were no differences in sex, age, preoperative New York Heart Association (NYHA) functional class, and comorbidities (Table 1).

**Table 1:** Baseline

	SR Group, *n* = 85	FB Group, *n* = 86	*Р* value
FED, n (%)	65 (76.5)	56 (65.1)	0.103
Forme fruste, n (%)	12 (14.1)	18 (20.9)	0.241
Barlow, n (%)	8 (9.4)	12 (14.0)	0.355
Male, n (%)	59 (69.4)	58 (67.4)	0.782
Age, years	57 (23–75)	54 (19–74)	0.092
Height, cm	173 (148–192)	175 (151;193)	0.079
Weight, kg	79.1 ± 14.6	77.1 ± 16.4	0.388
BMI, kg/m^2^	26.9 ± 4.3	25.4 ± 4.6	0.522
BSA, m^2^	1.94 ± 0.21	1.93 ± 0.24	0.801
NYHA I, n (%)	7 (8.2)	12 (14.0)	0.234
NYHA II, n (%)	22 (25.9)	29 (33.7)	0.263
NYHA III, n (%)	54 (63.5)	43 (50.0)	0.742
NYHA IV, n (%)	2 (2.4)	2 (2.3)	0.991
AF, n (%)	22 (25.9)	16 (18.6)	0.252
Paroxysmal, n (%)	2 (2.4)	2 (2.3)	0.991
Persistent, n (%)	5 (5.9)	3 (3.5)	0.459
Long-standing, n (%)	11 (12.9)	9 (10.5)	0.614
Permanent, n (%)	4 (4.7)	2 (2.3)	0.398
CAD, n (%)	10 (11.8)	4 (4.7)	0.090
Arterial hypertension, n (%)	46 (54.1)	42 (48.8)	0.490
Diabetes mellitus, n (%)	4 (4.7)	5 (5.8)	0.746
Moderate renal impairment, n (%)	1 (1.2)	1 (1.2)	0.993
Peripheral vascular disease, n (%)	5 (5.9)	3 (3.5)	0.459
Cerebrovascular disease, n (%)	5 (5.9)	4 (4.7)	0.719
LV EF, %	65.5 (49–80)	67.0 (51–89)	0.212
PA pressure, mm Hg	48.5 (29–80)	45.0 (29–94)	0.053

*SR* semirigid ring, *FB* flexible band, *BMI* body mass index, *BSA* body surface area, *NYHA* New York Heart Association functional class, *AF* atrial fibrillation, *CAD* coronary artery disease, *LV EF* left ventricle ejection fraction, *PA* pulmonary artery

### Outcome measures

The primary endpoint was freedom from moderate or severe mitral regurgitation (MR) recurrence. Secondary endpoints included survival, freedom from reoperations, and freedom from severe MR recurrence. The severity of MR was evaluated and defined in accordance with the recommendations [9]. Valve-related complications were evaluated and defined in accordance with the guidelines [10].

### Surgical techniques

Real-time two-dimensional/three-dimensional transesophageal echocardiography (TEE, Phillips iE33, Philips Ultrasound Inc., PA, USA) was performed after the induction of anesthesia for MV lesion estimation. Cold crystalloid cardioplegic solution (Custodiol® HTK Solution, Dr. Franz Köhler Chemie, Alsbach-Hahnlein, Germany) was used for myocardial protection with antegrade root flow.

The two most experienced surgeons in MV repair in our Institute performed the operations in the study. Surgeons were evenly split between the treatment groups. The surgical technique for MV repair was chosen according to the MV lesion (Table 2). The most common techniques were quadrangular (“sliding maneuver”) or triangular resection for posterior MV prolapse, and artificial chordal replacement (separate or loop technique) for anterior leaflet pathology. The minimally invasive approach through the right fourth intercostal space with femoral–femoral cannulation for arterial and venous lines was used in 29.4% (*n* = 25) and 31.4% (*n* = 27) of patients in the SR and FB groups, respectively.

**Table 2:** Intraoperative data

	SR Group, *n* = 85	FB Group, *n* = 86	*P* value
Approach:			
Conventional, n (%)	60 (70.6)	59 (65.1)	0.778
Minimally invasive, n (%)	25 (29.4)	27 (31.4)	0.778
CPB time, min	140 (110;179.5)	160 (122;206)	0.091
Cross clamp time, min:			
Conventional	92 (73;117)	101 (79;125)	0.230
Minimally invasive	116 (101;129)	123 (106;156)	0.171
Mitral valve analyze and intervention in certain patients			
А_1_ prolapse, n (%)	9 (10.6)	11 (12.8)	0.654
А_2_ prolapse, n (%)	23 (27.1)	33 (38.4)	0.115
А_3_ prolapse, n (%)	13 (15.3)	19 (22.1)	0.254
P_1_ prolapse, n (%)	6 (7.1)	12 (13.9)	0.142
P_2_ prolapse, n (%)	59 (69.4)	68 (79.1)	0.119
P_3_ prolapse, n (%)	17 (20.0)	27 (31.4)	0.088
AMVL prolapse, n (%)	31 (36.5)	40 (46.5)	0.183
PMVL prolapse, n (%)	73 (85.9)	74 (86.1)	0.975
DMVL prolapse, n (%)	19 (22.4)	28 (32.5)	0.135
Chordae rupture, n (%)	64 (75.3)	53 (61.6)	0.055
AMVL resection, n (%)	1 (1.2)	4 (4.7)	0.178
PMVL resection, n (%)	37 (43.5)	49 (56.9)	0.079
AMVL neochordae, n (%)	22 (25.9)	29 (33.7)	0.263
PMVL neochordae, n (%)	30 (35.3)	27 (31.4)	0.589
Ring size, mm	32 (30;32)	34 (30;34)	0.456
Concomitant procedure			
Maze IV procedure, n (%)	18 (21.2)	14 (16.3)	0.412
CABG, n (%)	10 (11.8)	4 (4.7)	0.090
TV repair, n (%)	32 (37.6)	24 (27.9)	0.175
SAM-syndrome, n (%)	0	1 (1.2)	0.319
Intraoperative TEE			
Depth of coaptation, mm	9 (7;11)	6 (5;8)	0.006
Peak MV gradient, mm Hg	8.0 (6.7;10.9)	6.4 (4;9)	<0.001
Mean MV gradient, mm Hg	3 (2.4;4)	2 (2;3.9)	<0.001

*SR* semirigid ring, *FB* flexile band, *AMVL* anterior mitral valve leaflet, *PMVL* posterior mitral valve leaflet, *DMVL prolapse* dual (anterior and posterior) mitral valve leaflet prolapse, *CABG* coronary artery bypass grafting, *TV* tricuspid valve, *CPB* cardiopulmonary bypass, *TEE* transesophageal echocardiography

All mitral valve repairs were performed with flexible bands (“C Flex”, CardiaMed, Penza, Russia) or complete rings (“D Ring”, CardiaMed, Penza, Russia), which routinely use since 2005. The ring size was selected according to the size of the anterior leaflet and the intertrigonal distance. In Barlow’s cases, 36–40 mm rings were used. Flexible bands were implanted along the posterior mitral semicircle with an extension to both commissures for 3–5 mm. Valve competence was assessed intraoperatively by saline test and “ink-test” (symmetrical closure line, coaptation depth ≥ 5 mm, absence of leaflet prolapse and regurgitation jet).

After bypass weaning, patients were followed-up with TEE. In cases with residual MR ≥ grade 2, cardiopulmonary bypass was re-established, and the valve was re-repaired or replaced; residual MR grade 1 (mild) was left alone. Intraoperative data and concomitant procedures are shown in Table 2.

### Patient follow-up

All patients underwent TTE evaluation before discharge. In total, 170 patients were discharged and followed-up periodically by cardiologists and surgeons. After discharge, examinations were scheduled annually. When annual clinic visits were unavailable, follow-up was performed by contact with the referring cardiologist, the patient or their family. Anticoagulation therapy with an international normalized ratio (INR) target in the range of 2.5–3.0 was prescribed for all patients after surgery. The decision to stop anticoagulation therapy with Coumadin was based on echocardiography (normal left ventricle function, presence of atrial contractility) and Holter data (absence of atrial fibrillation, flutter, tachycardia) after 3 months. Echocardiograms obtained from outside physicians were re-analyzed at our Institute by the most experienced echocardiographers. Clinical follow-up was completed in 165 patients (97.1%), and five patients were lost to follow-up. Follow-up echocardiography was performed 6–12 months after the operation and every year thereafter. Seventy-nine (94.0%) of the 84 discharged patients in the SR group and 78 (90.7%) of the 86 patients in the FB group underwent follow-up TTE. The mean duration from the operation to echocardiographic follow-up was 20.2 (95% confidence interval [CI], 18.6–21.8) months. The mean clinical follow-up period was 24.7 (95% CI, 23.5–27.0) months.

### Statistical analysis

Assuming 5% significance (two sided), 80% power, event rates of 20 and 5% in the complete ring and flexible band groups, respectively, and a hazard ratio for significant MR recurrence of 0.23 and expected 5% withdrawal rate, the sample size (total *n* = 170 in both groups) was calculated using log-rank test (Freedman method) of freedom from MR recurrences in two groups. Data are presented as mean ± SD or median with range. The variables of the two groups were compared using the unpaired *t* test for continuous variables with normal distribution or the Mann-Whitney *U* test for other distributions. To analyze the risk factors of early postoperative complications, we reviewed preoperative and intraoperative variables (Tables 1, 2, 3); a multivariate logistic regression model was used to calculate odds ratios (ORs). Estimates of survival and event-free survival for reoperation and the recurrence of significant MR were calculated using the Kaplan-Meier method and are reported with 95% CIs. Patients were censored at the time of reoperation or at the time of death. Estimates are reported with their standard errors. Comparison of the curves was established by using the log-rank test for mid-term results. To analyze risk factors of late mortality, reoperation, and MR recurrence, we reviewed preoperative and intraoperative variables (Tables 1, 2, 3); multivariable Cox proportional hazard regression models were used to calculate HRs. HRs and 95% CIs were calculated. The inclusion criterion for the multivariable model was *P* value ≤0.2) in the univariable analysis. The significance level in the final “multivariable” model assessed as the 0.05. Stata/MP for Windows v. 13.0 (StataCorp. 2013. *Stata Statistical Software: Release 13*. College Station, TX: StataCorp LP.) was used for the statistical analysis.

**Table 3:** Echocardiography data

	SR Group, *n* = 85					FB Group, *n* = 86					Group comparison, *P* value		
	Baseline	At discharge	*Р* value at discharge	Follow up, *n* = 79	*P* value	Baseline	At discharge	*P* value at discharge	Follow up, *n* = 78	*P* value	Baseline	At discharge	Follow up
RA, cm	5.4 (4.1–8.9)	4.9 (4.0–7.9)	<0.001	4.8 (3.6–7.8)	0.499	5.3 (3.3–8.5)	4.8 (3.7–6.8)	<0.001	4.8 (3.0–7.5)	0.805	0.155	0.187	0.204
LA, cm	6.1 (4.3–9.6)	5.2 (4.4–8.2)	<0.001	5.1 (3.5–7.9)	0.460	5.7 (4.4–9.5)	5.1 (4.0–7.2)	<0.001	4.9 (3.2–7.2)	0.070	0.070	0.067	0.291
S МО (Doppler) cm^2^	3.8 (2.6–6.9)	3.3 (2.4;7.6)	<0.001	3.2 (1.9–5.7)	0.806	4.1 (2.8–9.8)	3.3 (2.6–4.9)	<0.001	3.3 (1.8–4.8)	0.054	0.062	0.202	0.699
Peak MV pressure gradient, mm Hg	8.7 (3–24)	8.8(2.7–17.0)	0.684	8.5 (3.9–17)	0.206	7.9(3.0–22.0)	7.0 (2.1–15.2)	0.027	6 (2.1–18.0)	0.212	0.680	<0.001	<0.001
Mean MV pressure gradient, mm Hg	2.8 (1–8)	3.0 (1.0–8.0)	0.236	3.7 (1.3–8.0)	0.288	2.6 (1.0–7.0)	2.7 (0.9–5.7)	0.872	2.8 (0.6–6.8)	0.232	0.985	0.002	0.001
Severe MR, n (%)	85 (100)	0	<0.001	7 (8.9)	0.023	86 (100)	0	<0.001	1 (1.3)	1.0	1.0	1.0	0.031
Total moderate or severe MR, n (%)	85 (100)	5 (5.9)	<0.001	12 (15.2)	0.023	86 (100)	4 (4.6)	<0.001	7 (9.0)	0.248	1.0	0.719	0.343
LV EDD, cm	5.78 ± 0.59	5.03 ± 0.53	<0.001	4.82 ± 0.67	0.135	5.78 ± 0.74	5.04 ± 0.52	<0.001	4.83 ± 0.47	0.017	0.962	0.874	0.908
LV ESD, cm	3.49 ± 0.55	3.36 ± 0.49	0.055	3.3 ± 0.7	0.731	3.51 ± 0.71	3.35 ± 0.58	0.783	3.15 ± 0.46	<0.001	0.815	0.891	0.263
LV EDV, ml	172.5 ± 42.0	120.9 ± 32.6	<0.001	111.2 ± 42.7	0.487	169.0 ± 50.0	123.4 ± 32.3	<0.001	105 ± 23.1	0.001	0.624	0.618	0.375
LV ESV, ml	53.0 (19–145)	44.5 (22–135)	<0.001	35 (18–102)	0.130	50.5 (16–146)	45 (20–140)	0.165	38 (18–154)	<0.001	0.127	0.360	0.808
LV EF, %	65.5 (49–80)	61 (35–76)	<0.001	61 (35–87)	0.518	67.0 (51–89)	59 (35–78)	<0.001	65 (36–78)	<0.001	0.212	0.247	0.043
PA systolic pressure, mm Hg	48.5 (42;56)	36 (29–46)	0.003	34.5 (20–68)	0.080	45 (39;54.5)	36.5 (25–49)	<0.001	29.5(8–48)	0.833	0.0n3	0.416	<0.001

*SR* semirigid ring, *FB* flexible band, *RA* size of right atrium, *LA* size of left atrium, *S MO* mitral orifice area, *MV* mitral valve, *MR* mitral regurgitation, *LV* left ventricle, *EDD* end diastolic diameter, *ESD* end systolic diameter, *EDV* end diastolic volume, *ESV* end systolic volume, *EF* ejection fraction, *PA* pulmonary artery

### Availability of data and materials

The data that support the findings of this study are available from Meshalkin National Medical Research Center but restrictions apply to the availability of these data, which were used under license for the current study, and so are not publically available. Data are however available from the authors upon reasonable request and with permission of Meshalkin National Medical Research Center.

## Results

Overall repair rate was 95%. The mean (median) cross clamp time did not differ between the SR and FB groups. The subgroup analysis of variances by the surgical approach revealed no differences in cross clamp time between the minimally invasive (25 and 27 patients, respectively, *p* = 0.171) and median sternotomy subgroups (60 and 59 patients, respectively, *p* = 0.230).

The incidence of systolic anterior motion (SAM) syndrome was observed in 1 patient in the FB group. Conservative management was not effective and SAM was successfully treated using posterior MV leaflet folding technique. Two patients from SR group were re-repaired due to residual moderate MR revealed by intraoperative TEE.

Intraoperative TEE control revealed that MV coaptation depth was significantly higher in the SR group than the FB group (9 [7–11] vs. 6 [5–8] mm; *p* = 0.006); however, the FB group had a lower rate of peak and mean transmitral pressure gradients (*p* < 0.001; Table 2).

There were no early (at 30/60/90 days) deaths. One 65 years old man with p3 prolapse and coronary artery disease who was underwent triangular posterior leaflet resection and concomitant CABG in the SR group died after 6 months of hospital stay because of severe multiple organ failure. Two patients (one from each group) with severe LV systolic dysfunction in early postoperative period required extracorporeal life support with complete recovering after 7 days.

The mean intensive care unit stay was 2 days in both groups (*p* = 0.453). Ventilation and inotropic support time also did not differ between the groups (Table 4). There were no significant differences in terms of heart failure, prolonged ventilation or requirement of extracorporeal life support. Electrical cardioversion for atrial fibrillation paroxysm was required in 7 (8.2%) and 3 (3.5%) cases in the SR and FB groups, respectively (*p* = 0.186). Five patients (SR group, 4 vs. FB group, 1; *p* = 0.169) were re-explored for bleeding on the first postoperative day. Pacemaker implantation rates were 5.9% (due to sinus node dysfunction, 3 patients; complete AV-conductance disturbances, 2 patients) and 4.7% (due to sinus node dysfunction, 3 patients; complete AV-conductance disturbances, 1 patient) in the SR and FB groups, respectively (*p* = 0.719).

**Table 4:** Early (30-days) results

	SR Group, *n* = 85	FB Group, *n* = 86	*Р* value
Hospital mortality^a^, n (%)	1 (1.2)	0	0.313
ICU stay, days	2 (1–14)	2 (1–14)	0.453
Ventilation time, h	7 (0–49)	5 (1–41)	0.068
Inotropic support, h	10 (0–151)	14 (0–105)	0.591
Heart failure, n (%)	23 (27.1)	19 (22.1)	0.451
Prolonged ventilation, n (%)	14 (16.5)	15 (17.4)	0.866
ECLS, n (%)	1 (1.2)	1 (1.2)	0.993
AF Early paroxysm, n (%),	39 (45.9)	30 (34.9)	0.143
Electrical cardioversion, n (%)	7 (8.2)	3 (3.5)	0.186
Valve-related complications, n (%):			
MI, n (%)	5 (5.9)	3 (3.5)	0.459
TIA, n (%)	4 (4.7)	0	0.042
Stroke, n (%)	0	1 (1.2)	0.319
AKF, n (%)	5 (5.9)	4 (4.7)	0.719
IE, n (%)	1 (1.2)	1 (1.2)	0.993
Ring dehiscence, n (%)	1 (1.2)	0	0.313
Leak, n (%)	0	0	–
Thrombosis, n (%)	0	0	–
Other embolic events, n (%)	0	0	–
Structural dysfunction, n (%)	0	0	–
Bleeding, n (%);	9 (10.6)	6 (7.0)	0.404
Re-exploration for bleeding, n (%)	4 (4.7)	1 (1.2)	0.169
Lymphorrhea, n (%)	0	1 (1.2)	0.319
Pacemaker implantation, n (%)	5 (5.9)	4 (4.7)	0.719
Pleural effusion, n (%)	14 (16.5)	20 (23.2)	0.266
Deep sternal infection, n (%)	0	0	–
Superficial infection, n (%)	1 (1.2)	1 (1.2)	0.993
Hospital stay, days	17 (9–178)	17 (7–36)	0.455

*SR* semirigid ring, *FB* flexible band, *ICU* intensive care unit, *ECLS* extracorporeal life support, *AF* atrial fibrillation, *MI* myocardial infarction, *TIA* transient ischemic attack, *AKF* acute kidney failure, *IE* infective endocarditis

^a^hospital death occurred 6 months after surgery

There were no significant between-group differences in major valve-related complications. There were no cases of thromboembolic events, leakage, or structural dysfunction. There were 4 cases of transient ischemic attack with reversible neurologic deficits in the SR group versus none in the FB group (*p* = 0.042).

The median length of hospital stay after MV repair in the SR and FB groups was 17 (9–178) and 17 (7–36) days, respectively (*p* = 0.455, Table 4). The preoperative independent predictor of heart failure was left ventricular ejection fraction (LVEF) (OR, 0.94; 95% CI, 0.9–0.98); of myocardial infarction – cardiopulmonary bypass time with 1-min step (OR, 1.03; 95% CI, 1.01–1.1); and prolonged ventilation – preoperative NYHA functional class (OR, 2.6; 95% CI, 1.2–5.7).

### Echocardiographic results

All echocardiographic parameters significantly changed from the preoperative to immediate postoperative period at discharge, except transmitral pressure gradients and LV end-systolic diameter (LVESD) (Table 3). There were no significant between-group differences in changes in LV function and left and right atria remodeling; however, transmitral peak and mean pressure gradients were significantly lower in the FB group. The mitral orifice area did not differ between the groups. The TTE before discharge revealed that 5 (5.9%) and 4 (4.7%) patients in the SR and FB groups, respectively, had grade 2 residual MR (*p* = 0.719). Follow-up echocardiography data at 24 months are shown in Table 3. The repeated-measures analysis of variance in the FB group revealed significant changes in LV end-diastolic diameter (LVEDD), LV end-diastolic volume (LVEDV), and LV end-systolic volume (LVESV) at discharge; moreover, these parameters and LVESD were also decreased at 24 months of follow up. Meanwhile, the LVEF significantly decreased at discharge; however, it significantly increased during the follow-up. In the SR group, there were no significant differences in LV function changes at serial examinations. We also revealed higher transmitral pressure gradients in the SR group, including significant differences in systolic pulmonary artery pressure among patients with sinus rhythm, with favorable results in the FB group.

### Survival analysis

**Fig. 2:**
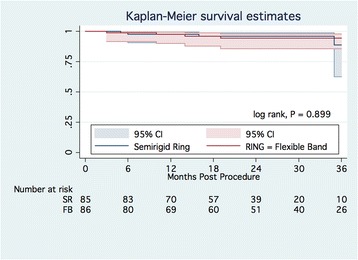
Kaplan-Meier survival estimates. Abbreviation: SR – semirigid ring; CI, confidence interval

At the latest follow-up, 158 patients were alive. There were 3 late deaths among the 84 patients in the SR group (mortality 3.6%), and 4 late deaths among the 81 patients in the FB group (mortality 4.9%). The causes of death in the SR group were endocarditis (1 case), malignancy (1 case), and unknown cause (1 case); two of them were regarded as cardiac-related deaths. The causes of death in the FB group were ischemic stroke (2 cases), acute myocardial infarction (1 case), and pulmonary edema (1 case), all of which were cardiac-related. The Kaplan-Meier survival rates at 2 years were 96.0 ± 2.3% (95% CI, 88.6–98.7%) and 94.3 ± 2.8% (95% CI, 85.5–97.9%) in the SR and FB groups, respectively (Figure 2) (log-rank, *p* = 0.899).

The Kaplan-Meier freedom from cardiac-related death at 2 years was 97.7 ± 2.0% (95% CI, 89.1–99.3%) and 94.3 ± 2.8% (95% CI, 85.5–97.9%) in the SR and FB groups, respectively (Additional file 1: Figure S1) (log-rank, *p* = 0.411). The multivariable Cox regression hazard model did not identify independent risk factors of late death.

### Clinical status

Before surgery, 65.9% and 52.3% of patients in the SR and FB groups, respectively, had an NYHA functional classification of III and IV. Most patients demonstrated significant improvement of functional capacity at the last follow-up (43.2% and 34.6% of 81 SR patients and 44.1% and 40.3% of 77 FB patients had an NYHA functional classification of I and II, respectively). A total of 57 (75%) and 61 (84.7%) patients presented with sinus rhythm in the SR and FB groups, respectively (*p* = 0.201).

### Thromboembolism and anticoagulation-related hemorrhage

There was no major bleeding case during follow-up. Two patients form each group had transient ischemic attack. In the FB group, 2 out of 3 patients who had stroke died. Two patients had atrial fibrillation and were kept on anticoagulation therapy, and another one was in sinus rhythm at the last follow up and was not anticoagulated. Central retina occlusion occurred in 1 case in each group. The overall freedom from thromboembolic events at 3 years after MV repair in the SR and FB groups was 94.7 ± 3.6% (95% CI, 80.6–98.7%) and 90.8 ± 3.7% (95% CI, 80.4–95.9%), respectively (log-rank, *p* = 0.416).

### Infective endocarditis

Three patients had infective endocarditis. Two of them were in the SR group, and one of them died because of pacemaker lead endocarditis and heart failure. The 2 patients who survived were treated with antibiotics alone. Freedom from infective endocarditis at 3 years was 97.3 ± 1.9% (95% CI, 89.4–99.3%) and 98.5 ± 1.5% (95% CI, 89.9–99.8%) in the SR and FB groups, respectively (log-rank, *p* = 0.573).

### Recurrence of mitral regurgitation

**Fig. 3:**
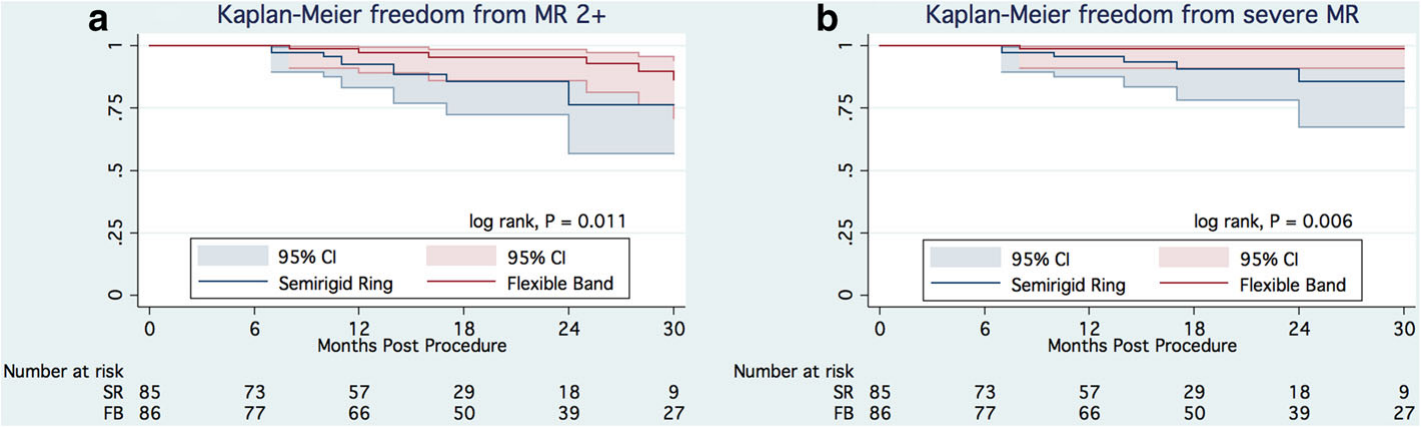
**a**. Kaplan-Meier freedom from moderate and severe mitral regurgitation. **b**. Kaplan-Meier freedom from severe mitral regurgitation. Abbreviation: MR, mitral regurgitation; SR – semirigid ring; CI, confidence interval

Moderate or severe MR was observed in 12 (15.2%) of 79 patients and 7 (9.0%) of 78 patients in the SR and FB groups, respectively (*p* = 0.343). Among them, 3 patients in the SR group underwent redo MV surgery. The Kaplan-Meier estimate for freedom from recurrence of moderate or severe MR at 2 years was 85.3 ± 5.0% (95% CI, 71.9–92.6%) and 95.3 ± 2.7% (95% CI, 85.9–98.5%) in the SR and FB groups, respectively, (log-rank test, *p* = 0.011) (Figure 3A). The multivariable Cox proportional hazard model identified residual MR before discharge as an independent risk factor of late recurrence of significant MR (HR, 4.1; 95% CI, 1.5–11.5) (Additional file 2: Table S1).

Among them, severe MR was observed in 7 (9.7%) and 1 (1.3%) patient(s) in the SR and FB groups, respectively. The Kaplan-Meier estimate for freedom from severe MR at 2 years was 84.8 ± 6.8% (95% CI, 65.1–93.8%) and 98.7 ± 1.3% (95% CI, 90.8–99.8%) in the SR and FB groups, respectively (log-rank test, *p* = 0.006) (Figure 3B). The multivariable Cox proportional hazard model identified residual MR before discharge as an independent risk factor of late recurrence of severe MR (HR, 8.4; 95% CI, 1.6–43.3).

### Reoperations

**Fig. 4:**
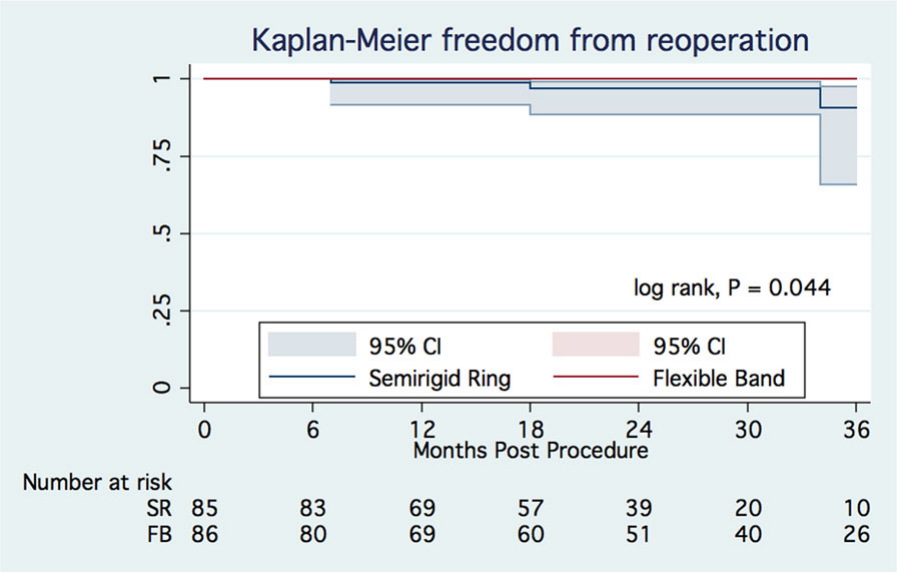
Kaplan-Meier freedom from reoperations. Abbreviation: SR – semirigid ring; CI, confidence interval

Reoperations were required in 3 patients in the SR group for partial ring dehiscence in all cases. The MV was re-repaired in 2 patients and replaced in 1 case. The Kaplan-Meier estimate for freedom from reoperation at 2 years of follow-up was 97.0 ± 2.1% (95% CI, 88.4–99.3%) and 100% in the SR and FB groups, respectively (Figure 4) (log-rank, *p* = 0.044). The Cox regression hazard model did not identify any preoperative or intraoperative risk factor of late reoperations.

## Discussion

In the late 1960’s, Carpentier developed annuloplasty rings, considered the “gold standard” for the surgical treatment of MR [11]. Subsequent studies showed that the MV annulus continually changes size and shape during the cardiac cycle [12–14]. This led to the development of a flexible ring that could conform to the physiologic changing annular shape [15, 16]. However, there is controversy regarding the optimal mechanical characteristics of annuloplasty rings and the use of rigid rings or flexible bands in degenerative MV disease [5, 17].

David et al. [18] conducted one of the first randomized studies comparing the rigid ring (13 patients) and flexible ring (12 patients). The results showed significantly better LV systolic function and stroke volume/LVEDV index in the flexible group and no significant reduction in LVESV and LVESD in the rigid ring group. However, the clinical outcomes of these 2 ring types have not been reported. In the present study, there were similar results in LV changes with no significant differences between the SR and FB groups intermediately and 2 years postsurgery. However, only the FB group showed a significant reduction in LVEDD, LVESD, LVEDV, and LVESV (when comparing postoperative values and those 2 years postsurgery).

Chang et al. conducted the largest randomized trial (363 patients) comparing the Carpentier-Edwards rigid ring and flexible Duran annuloplasty ring [19]. The mean duration from the operation to follow-up echocardiography was 26.7 ± 24.1 months. LVEF, LVESD, and LVEDD parameters changed significantly at serial examinations in both groups with no significant difference between the two ring types. There were no significant differences in survival, reoperation rates, and recurrences of significant MR at a mean follow-up of 46.6 ± 32.6 months. However, their study was limited by the heterogeneous MR etiology, the long recruitment period, and the fact that the echocardiography parameters were not presented at separate time points, which may have caused bias.

Shahin et al. [20] conducted a randomized study comparing the Carpentier-Edwards rigid Classic and semi-flexible Physio rings (mean follow up, 5.1 years). There were no between-groups differences in terms of mortality, morbidity, and LV function. However, an unexplained 16% difference in mortality was considered clinically important.

At least 3 randomized studies did not demonstrate the clinical benefits of flexible or rigid types of annuloplasty devices [18–20]. However, one of them [18] suggested better LV systolic function with flexible rings. Similar results were reported by Okada et al. [21], and concern that rigid rings could restrict LV wall motion was raised.

In a prospective echocardiographic study by Unger-Graeber et al. [22], no significant difference was shown between the Carpentier rigid ring and flexible Duran ring in terms of transmitral velocity and pressure gradient. In contrast, our study showed higher transmitral pressure gradients in the SR group.

After David et al. criticized the rigid ring for impaired LV function [18] and Kreindel et al. [23] reported the potential risk of SAM syndrome after rigid ring annuloplasty, Carpentier et al. introduced a new concept of mitral annuloplasty with the semirigid prosthetic ring, which combines remodeling and flexibility [24]. At mid-term follow-up between 6 and 18 months, 93.2% of 94 followed-up patients were free from MR recurrence with a transmitral pressure gradient at 3.55 ± 1.93 mmHg. Thereafter, excellent mid-term results with semirigid rings have been reported by other investigators [25, 26]. Since then, the concept of a semirigid ring has gained popularity, and the semirigid ring (instead of the rigid ring) has been successfully adopted in mitral annuloplasty for degenerative MV disease. Flexible posterior annuloplasty bands have also shown high effectiveness in preserving mitral annulus flexibility and provide good mid-term and long-term durability [4, 27, 28].

The semirigid ring and flexible ring have not been sufficiently compared. An animal randomized study [29] showed that LV function was not altered with either flexible or semirigid ring annuloplasty. A Japanese, retrospective, propensity score matched study [30] evaluated intermediate echocardiography results only. The overall cohort’s LVEF decreased during the first week after surgery and then recovered gradually at 6 months and 1 year; LVEDD abruptly decreased and LVESD minimally decreased at the first week postoperatively, then gradually decreased at 6 months and later stabilized. There were no significant between-group differences in LVEF, LVEDD, and LVESD. They suggested that the semirigid ring might prevent LV impairment compared with the rigid ring. However, their study was limited by the retrospective design, low rate of followed-up patients, and difference in annuloplasty devices used in the flexible group.

Previously was shown that experience in mitral valve repair is an important determinant of operative efficiency and late survival [31], in our study both surgeons are well experienced and evenly split between groups. However, our results seem different to Castillo JG report [32]. We assume that limited experience in Barlow valves disease was influenced for our results. In this view complex mitral valve cases should be consolidated and addressed to one surgeon. It might be helpful to use scoring system [33] to allow stratification of complexity for degenerative mitral valve repair for improving results and develop local expertise.

We examined the immediate and mid-term results of patients with degenerative MV disease who underwent primary MV repair with a complete semirigid ring or flexible posterior annuloplasty band. Both groups had comparable early clinical results; however, the SR group had better coaptation depth, while the FB group demonstrated significantly lower transmitral pressure gradients. At serial echocardiographic examinations, only the FB group showed significant LV remodeling. Consistent with previous studies, there were no between-group differences in overall survival, freedom from cardiac-related death, and follow-up LV remodeling. Our study is the first to show the superiority of the flexible posterior band over the semirigid complete ring in terms of freedom from recurrences of significant and/or severe MR and risk of MV reoperation.

## Conclusion

The present study is limited by the 2-year follow-up period and single-center design. Important limitation is low number of patients at late risk. Further study with a longer follow-up is warranted. In conclusion, patients with degenerative MV disease may benefit from valve repair with flexible bands. Residual MR is an independent risk factor of late insufficiency recurrence.

## Additional file

Additional file 1: Figure S1. Kaplan-Meier freedom from cardiac-related death. Abbreviation: CI, confidence interval. (TIFF 1293 kb)
Additional file 2: Table S1. Cox proportional hazard model for recurrence of significant MR. (DOCX 15 kb)

## Abbreviations

CI: Confidence interval
HR: Hazard ratio
ITT: Intention-to-treat
LV: Left ventricle
LVEDD: Left ventricle end-diastolic diameter
LVEDV: Left ventricle end-diastolic volume
LVEF: Left ventricular ejection fraction
LVESD: Left ventricle end-systolic diameter
LVESV: Left ventricle end-systolic volume
MR: Mitral regurgitation
MV: Mitral valve
NYHA: New York Heart Association
OR: Odds ratio
SAM: Systolic anterior motion
TEE: Transesophageal echocardiography
