# Supplementary figures and images for: Mid-term results of mitral valve repair using flexible bands versus complete rings in patients with degenerative mitral valve disease: a prospective, randomized study
Source: J Cardiothorac Surg. 2017 Dec 13;12:113. doi: 10.1186/s13019-017-0679-0 (PMC5729509; doi:10.1186/s13019-017-0679-0)

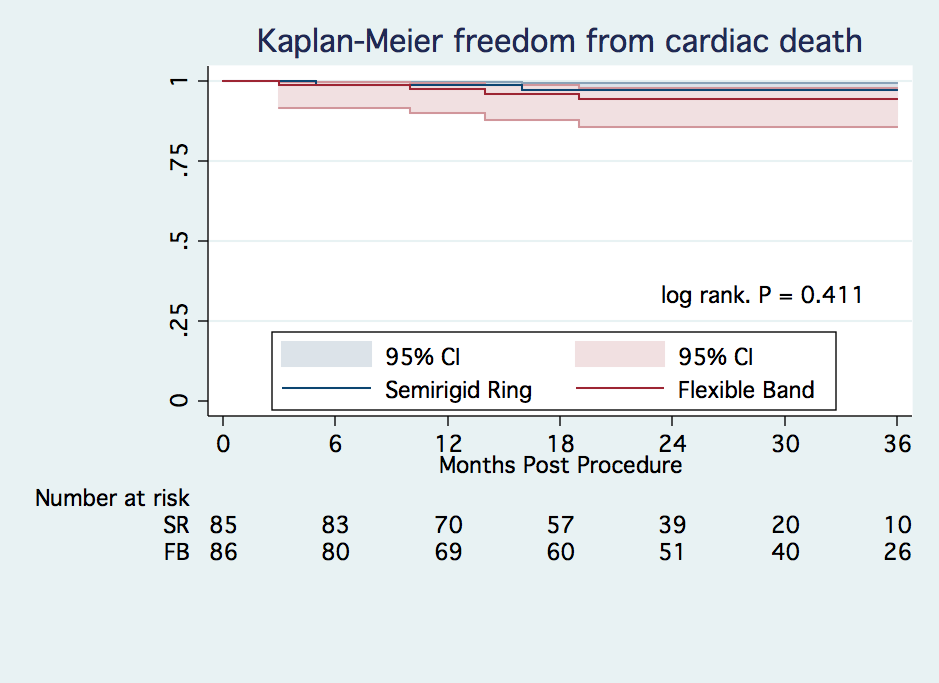

Supplement: Additional file 1: Figure S1. — Kaplan-Meier freedom from cardiac-related death. Abbreviation: CI, confidence interval. (TIFF 1293 kb) [file 13019_2017_679_MOESM1_ESM.tif]
