# Supplementary material for: Mid-term results of mitral valve repair using flexible bands versus complete rings in patients with degenerative mitral valve disease: a prospective, randomized study
Source: J Cardiothorac Surg. 2017 Dec 13;12:113. doi: 10.1186/s13019-017-0679-0 (PMC5729509; doi:10.1186/s13019-017-0679-0)
Supplement: Additional file 2: Table S1. — Cox proportional hazard model for recurrence of significant MR. (DOCX 15 kb) [file 13019_2017_679_MOESM2_ESM.docx]

**Additional file 1: Table S1. Cox proportional hazard model for recurrence of significant MR.**

| Risk factor | One variable model | | Multivariable model; χ^2^ =24.7; P < 0.001 | |
| --- | --- | --- | --- | --- |
|  | HR (95% CI) | Р value | HR (95% CI) | Р value |
| Residual MR at discharge | 5.0 (2.0-12.5) | 0.001 | 4.1 (1.5-11.5) | 0.008 |
| CAD | 1.9 (1.1 – 3.2) | 0.010 | 1.5 (0.8-2.5) | 0.176 |
| Systolic PA pressure, step 1 mm Hg | 1.03 (1.01-1.06) | 0.041 | 1.01 (0.98-1.04) | 0.379 |
| Semirigid ring | 3.57 (1.52-8.33) | 0.004 | 5.0 (2.0-12.5) | 0.001 |
| Barlow disease | 1.89 (0.24-12.54) | 0.788 | - | - |
| Ring size | 1.1 (0.93-1.21) | 0.371 | - | - |
| Depth of coaptation | 0.4 (0.09-11.4) | 0.645 | - | - |

HR – hazard ratio, MR – mitral regurgitation, CAD – coronary artery disease, PA – pulmonary artery
